# General guidelines for biomedical software development

**DOI:** 10.12688/f1000research.10750.2

**Published:** 2017-07-12

**Authors:** Luis Bastiao Silva, Rafael C. Jimenez, Niklas Blomberg, José Luis Oliveira

**Affiliations:** 1 BMD Software, Aveiro, Portugal; 2 ELIXIR Hub, Wellcome Trust Genome Campus, Hinxton, UK; 3 Institute of Electronics and Informatics Engineering of Aveiro, University of Aveiro, Aveiro, Portugal

**Keywords:** biomedical software, guidelines, software development, bioinformatics, Agile

## Abstract

Most bioinformatics tools available today were not written by professional software developers, but by people that wanted to solve their own problems, using computational solutions and spending the minimum time and effort possible, since these were just the means to an end. Consequently, a vast number of software applications are currently available, hindering the task of identifying the utility and quality of each. At the same time, this situation has hindered regular adoption of these tools in clinical practice. Typically, they are not sufficiently developed to be used by most clinical researchers and practitioners. To address these issues, it is necessary to re-think how biomedical applications are built and adopt new strategies that ensure quality, efficiency, robustness, correctness and reusability of software components. We also need to engage end-users during the development process to ensure that applications fit their needs. In this review, we present a set of guidelines to support biomedical software development, with an explanation of how they can be implemented and what kind of open-source tools can be used for each specific topic.

## Introduction

As an increasing number of scientific results are being generated from omics studies, new translational medicine applications and bioinformatics tools are needed to promote the flow of these results into clinical practice, i.e. the knowledge needs to be translated from the bench to the bedside, to foster development of new biotechnological products and improve patients’ health. Biomedical informatics intends to support the integration and transfer of knowledge across all major subject areas of translational medicine – from the study of individual molecules to the study of whole populations ^1^. Translational medicine brings together many areas of informatics, including bioinformatics, imaging informatics, clinical informatics and public health informatics ^2, 3^. Bioinformaticians, translational researchers and computational biologists identify the molecular and cellular components that can be targeted for specific clinical interventions and treatments for specific diseases. Imaging informatics also plays a significant role in understanding pathogenesis and identifying treatments at the molecular, cellular, tissue and organ level. Richer methods to visualize and analyse imaging data are already being investigated and developed ^4^. Other techniques such as text and data mining have been applied to clinical reports. Additionally, translational research teams need to focus on decision support, natural language processing (NLP), standards, information retrieval before applying these techniques to the electronic health records.

The biomedical informatics landscape is pushing for the development of more professional and easy-to-use software applications, in order to address the pressing need to translate research outcomes into clinical practice. To accomplish this, solid software engineering approaches must be adopted. Despite being a relatively young discipline, biomedical informatics has evolved at an impressive rate, constantly creating new software solutions and tools. However, due to their multidisciplinary nature, it is often difficult for individual studies to gather solid knowledge in their various fields. This problem has been flagged by several authors, who have proposed general competences that undergraduate students should acquire ^5, 6^. These competences can be obtained through introducing complementary courses, such as software programming, in existing curricula, or by creating new academic degree courses ^7^. While these strategies have resulted in many new and successful graduates, the right balance between looking for strong expertise in a single topic, or medium expertise in many topics, is not always easy to find. Nonetheless, it is important to address that there is a clear difference when software developers work for an academic thesis or project, compared to working in software companies. The academia projects are mostly frequent focused on the scientific novelty, while companies are more focused on achieving concrete results for the market needs. In both scenarios, software development methodologies need to be taken at distinct levels of complexity ^8^.

Many researchers without training in software engineering have found themselves faced with the intricate task of building their own software solutions. Moreover, researchers and clinicians typically perceive software development as an auxiliary task to serve science, rather than a central goal ^9^. The result is sometimes code-difficult and costly to maintain and re-use. This software dependency is indeed a problem across all science, where concerns about the reproducibility of research have raised the need for robust, open access and open source software ^10, 11^. The development of software projects requires effective collaboration between users and software developers, and also between the users themselves.

Another common drawback of current bioinformatics and clinical applications is the lack of user-friendly interfaces, making them difficult to use and navigate. User-centered design has also been proposed as a way to minimize this problem ^12^. The development of open source solutions has promoted software quality in the field, since it encourages public review, reuse, correction and continuous extension ^13^.

In concrete for bioinformatics, most of the software is written by researchers who use it for their own individual purposes, a process long-identified as end-user programming ^14^. However, these “new” programmers face many software engineering challenges, such as making decisions about design, reuse, integration, testing, and debugging ^15^. Several authors have tried to introduce software engineering approaches in bioinformatics programming to address this problem. Hastings *et al.* ^16^ compiled several recommendations that should be used to ensure the usability and sustainability of research software. Most of these suggestions are part of fundamental programming principles; e.g. keep simple, avoid repetitions, avoid spaghetti code. By examining a group of software projects, Rother *et al.* also identified a set of techniques that facilitate the introduction of software engineering approaches in academic projects ^17^. This work, which came from the authors’ own experience in conducting software projects, provided readers with a toolbox consisting of several steps, starting with traditional ones such as user stories and CRC cards. In a more specific study, Kamali *et al.* discussed several software testing methodologies that can be used in bioinformatics, such as simulators, testing in operational environment and cloud based software testing ^18^. Artaza *et al.* proposed 10 metrics for life science software development, identified as the most relevant by a group of experts ^19^. They include topics such as version control and software distribution or continuous integration. In a similar approach, Wilson *et al.* ^20, 21^ described a set of “good enough” principles that should be followed to better organize scientific computing projects, starting at the data gathering phase and continuing up to the writing of the manuscript.

This paper leverages on the experience of the MedBioinformatics project, which primary aim is to develop integrative bioinformatics tools and software applications useful and autonomously usable by translational scientists and clinical practitioners. We present a set of recommendations for biomedical software development, with an explanation of how they can be implemented and what kind of open-source tools can be used for each specific topic. These recommendations can be adopted in any kind of software development, from user-interface applications up to scripts developed to support biology and clinical research, which are very often ignored from the software development point of view.

## Why should we care about software development recommendations?

Many research organizations and teams can create biomedical software, but far too often, they are not sufficiently developed to be used by most clinical researchers and practitioners, because they are incomplete, lack user-friendly interfaces and software maintenance is not guaranteed after project completion. So, the main question we asked ourselves was how to ensure that the biomedical software development process in research institutes remained reliable and repeatable without them having to undertake major organizational changes.

Developing high quality biomedical software that accomplishes end-users’ expectations implies following a minimal set of software engineering guidelines. We propose the following:

• Team and project management
• Tracking the development process
• Software integration and interoperability
• Test-Driven Development (TDD) and continuous integration (CI)
• Documentation
• Software distribution
• Licensing

**Figure 1.**
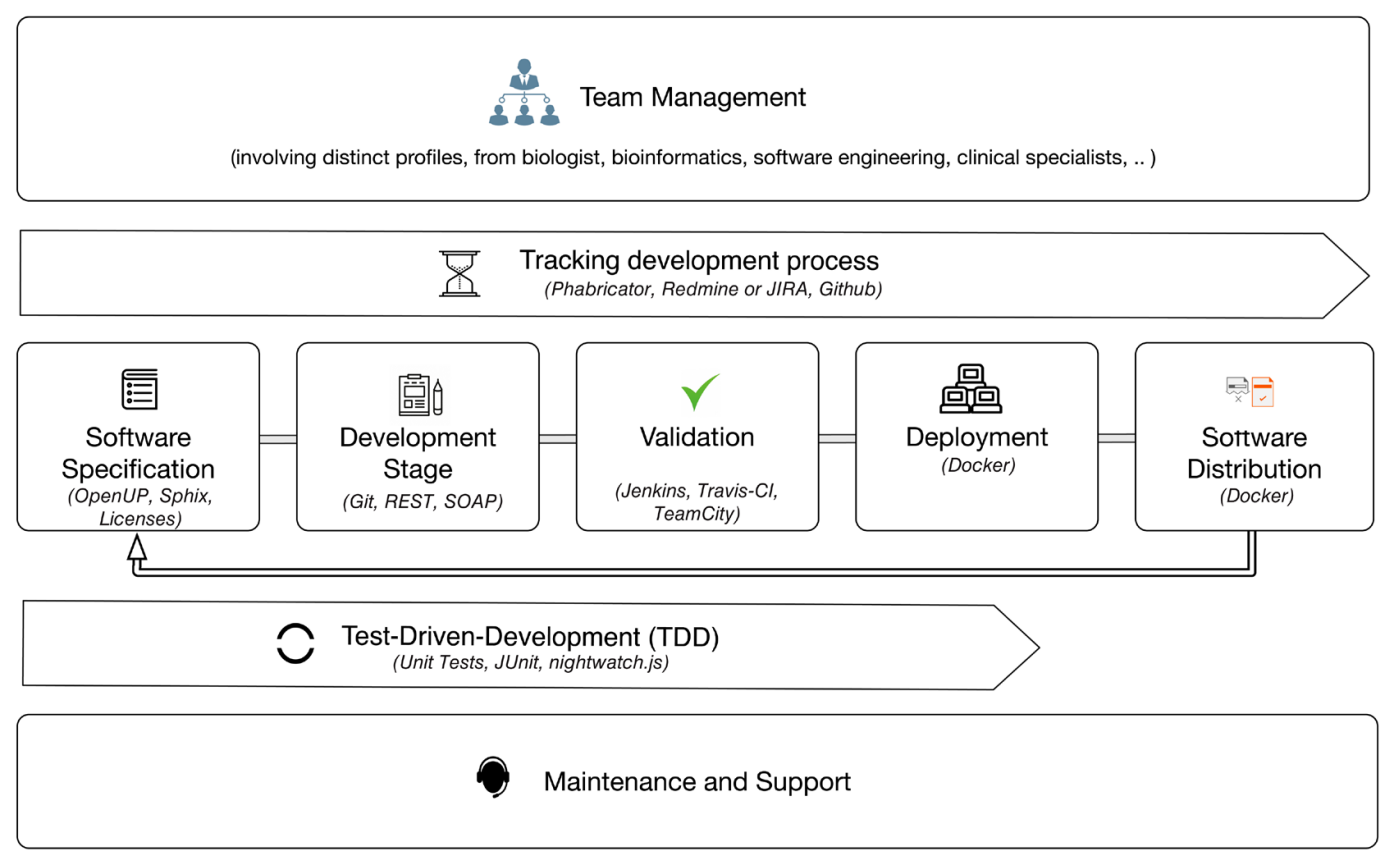
Software development process: including the several stages of the process.

Figure 1 presents a software development process that is following this general set of key steps. The first step, team and project management allows team members to keep track of group tasks and schedules, and be involved in development decisions. This encourages involvement of other users besides developers, who can point out missing features, give feedback and report bugs, helping communication between the whole team. Tracking the software development process consists of a combination of technologies and practices mostly used for source code management, but applicable to other collaborative tasks such as writing papers, product documentation, web site content, internal guidelines, and many more. Next, we have a cyclical pipeline between software integration and interoperability, which starts with the software specification phase and proceeds to the distribution phase, consisting of development, validation and deployment stages. The licensing of the software is one step that should be defined as early as possible, because during the development process it is often needed to include third-party dependency libraries, and the licenses should be compatible.

This test-driven development process can be used throughout the entire workflow, so that each unit is tested and the components’ integration is validated. Moreover, the documentation of each software module is important, and should be updated during all development phases. Finally, after the software application is distributed, appropriate maintenance and support is needed to assure end-users can rely on someone to handle their requests and help solve any problems.

To help the reader navigate through each of the following guidelines, we have divided each one into three sub-sections:

1) A summary that describes what it is intended for

2) The process description that explains what benefits it provides

3) Examples of tools and services that help to implement the guideline.

### Team and project management

**Summary**:

Team and/or project management tools are essential for many organizations, to help in planning and organizing teams, tasks, and schedules. Implementing them during software development allows teams to stay synchronized about task scheduling and milestones, and helps track individual and general progress, identifying difficulties early on so that the necessary adjustments can be made. There are various software applications available that manage the development process; they typically include a variety of features for planning, scheduling, controlling costs, managing budgets, allocating resources, collaborating, and making decisions.

**Process description**:

Tracking and organizing the development process typically involves the following main features:

• Task management – To prioritize what functionality is developed over the different phases of project. It is often provided as a graphical user interface tool that uses the *drag and drop* functionality to facilitate project management, such as Kanban boards – a method to visualize and manage the workflow, where one can move the tasks between different phases;
• Code reviewing – This important practice is often used to support teams of multiple developers, despite also being very useful to track the progress of a single developer. These tools allow the code to be audited by providing differential views of code changes, normally web-based interfaces where reviewers/auditors inspect the code independently, from their own machines, as opposed to synchronous review sessions where authors and reviewers meet to discuss changes;
• Source code repositories – A source code repository is a web hosting facility to store and manage source code and which normally supports version control;
• Bug tracking – Keeps track of all defects and problems with the source code, using a predefined nomenclature to describe each issue.

The process typically also includes document repositories, wikis, discussion forums, time-tracking, Gantt mapping, file storage, calendars and versioning control.

The principles behind team and project management tools have been implemented in several software development methodologies, such as Lean and Agile, and are important aspects of Scrum methodology, Kanban and extreme programming (XP) ^22^. Here, team management relies on several types of meetings, such as sprint planning meetings, daily Scrum meetings, sprint review meetings, sprint retrospective meetings and backlog refinement meetings. The Scrum Master is responsible for planning what will be discussed, namely what has been performed in the last sprint and what are intended to be done for the next sprint - a sprint is a specific period in which a set of tasks need to be accomplished. Developers also need to be prepared to analyse their development process, and negotiate future plans and potential deadlines. While Agile methodologies can lead to too many meetings, it is highly recommended to meet periodically to coordinate the development process.

**Examples**:

Depending on the type of financial resources available, free or open source management applications can be adopted, installed locally or used as a service in the cloud. Some examples of management applications are: Phabricator, Redmine or JIRA, Github and Bitbucket.

### Tracking the development process

**Summary**:

A source control management system (SCM) provides coordination and management services between members of a software development team. It could be implemented in many different ways, and the most basic level, it could be a shared folder, and only the newest versions of files are available for use. In software programming, when there are several team members, the concept of branches is very important. Quite often, projects are only supported by a single researcher, but this is also very important for these small projects. To correctly support the concept of branch, more complex software is required.

**Process description**:

The more recent versions of SCMs allows developers to work simultaneously on the same file, merge changes with other developers’ changes and track and audit changes that were pull requested. Nowadays, SCMs often include components to assist the code revision and also to manage software process milestones and roadmaps.

**Figure 2.**
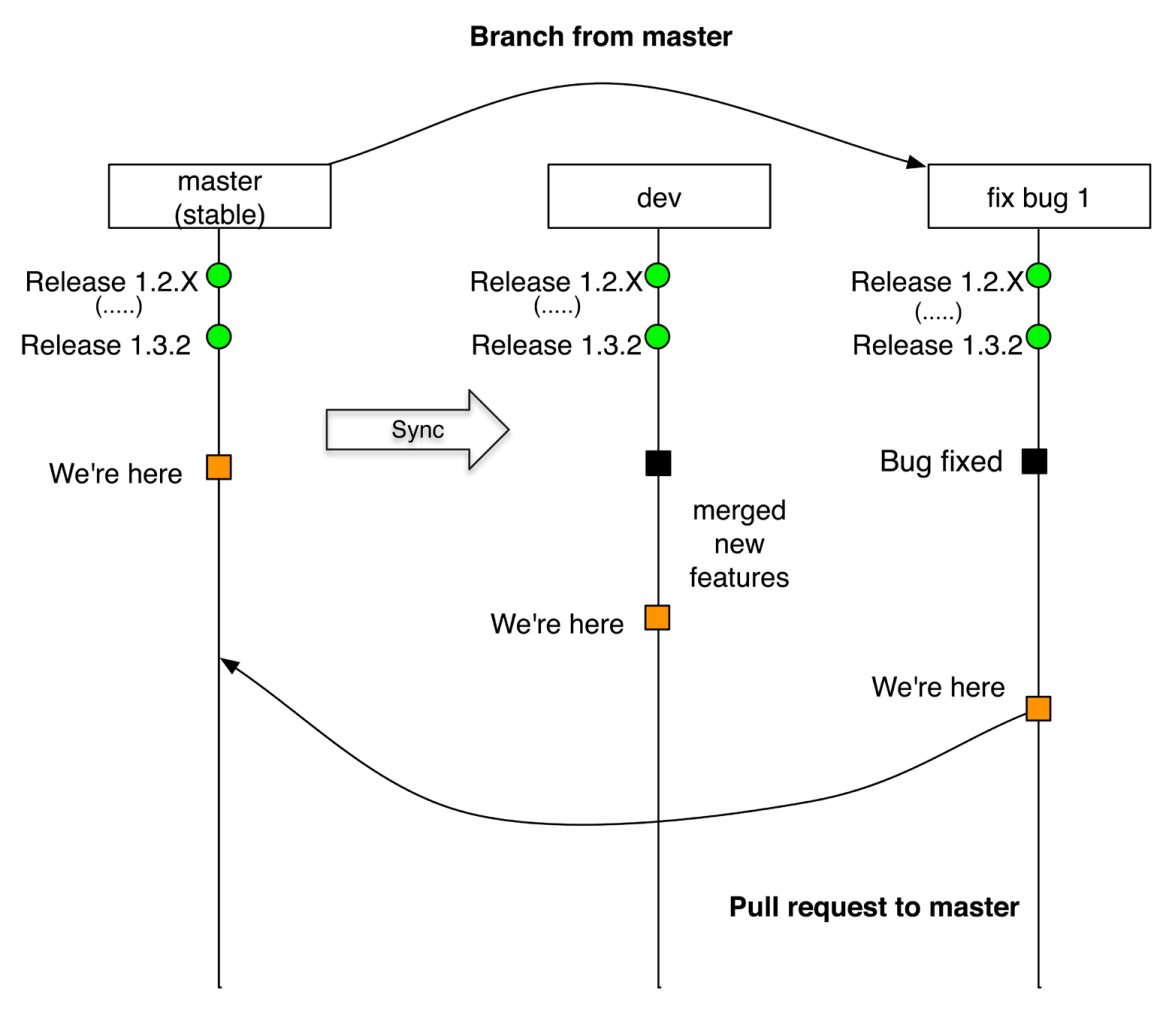
Example of a strategy for SCM workflow based on Git. It is an example of a bug fix branch from *master* branch and created a *pull request* with the changes against *master* branch.

There are several strategies to develop with Git, and in this section a short summary of Git Flow is presented – a well-known branch model developed by Vincent Driessen ^1^. The development process includes two branches: *master* and *dev*. Master will be the most stable branch. Only bug fixing can be merged in the *master* branch and the bug fix branches should always be pull requested to master. The *Dev* branch contains new features, and more unstable branches may be pull requested to this branch. This is where the developers are creating new features for the planned next releases of the software. Figure 2 shows an example of the bug fixing flux that occurs while a new branch is created from the master.

The process usually starts with an issue being reported, and after a decision has been made, it is assigned to a developer. Before going to production, it needs to pass internal tests overseen by an internal testing team. If the bug has been fixed according to requests, the case is closed, or a report is sent back to the developer with a new set of issues.

New features are developed according to users’ feedback. It is a complex task that often involves re-engineering the applications. This process may break some other features already in place. Thus, the new features are implemented in a development branch, passing through several analyses, tests and user feedback stages. Finally, release management is also performed within the SCM. Generally, it uses an incremental numbering schema to tag each version. In this way, it is always possible to track older versions and roll back to a previous version, which is mainly required to compare the behaviour of different versions.

The following best practices should be applied to software version control:

• Before committing, check for possible changes in the repository
• When committing a change to the repository, make sure the change reflects a single purpose (E.g. Fixing a bug, adding a new feature);
• If possible, try to create change sets linked to the issue tracker. Use the issue ID in the commit message;
• After merging, run the unit tests to ensure that the merge was successful;
• After creating a tag, do not commit to it any more. Visualize the tag as read-only. If it is necessary to resolve an issue in that specific version, create a branch from that tag and commit the changes to it;
• Try not to merge a large number of changes between the trunk and the branches. Use atomic commits;
• Make at least one commit per day with all the day’s work.

**Examples**:

Several version control systems (VCS) can manage code development, such as Git or Mercurial. Github or Bitbucket are some examples of ready-to-use SCM.

### Software integration and interoperability

**Summary**:

Software integration and interoperability with external systems is a very important requirement in the biomedical domain, due to the reusability of existing repositories, services, algorithms, components and even applications. Designing an application programming interface (API) is crucial in distributed system development, so that the final solution can interconnect and interoperate with other systems.

**Process description**:

A programming interface exposes part of a system behaviour, and it is sometimes difficult to implement when different platforms and programming languages are required. Since creating a new interface for each specific service could be tiresome and error-prone, it is often preferred to take a generic interface and express application-specific semantics to them. This is often a trade-off between performance, extensibility and stability of the API. To collaborate with specifying new semantics and the development of systems complying with such interfaces, Interface Description Languages (IDLs) emerged as formal definition languages for describing software interfaces, often coupled with facilities for documenting the API and generating consumer and provider code stubs for multiple platforms or programming languages.

Two of the most used types of API are SOAP ^2^ and REST ^23^:

• The Simple Object Access Protocol (SOAP) is an Internet protocol for messaging and remote procedure calls, using Extended Markup Language (XML) as the base message format and usually (although not necessarily) HTTP as the transport protocol. Web Service Definition Language (WSDL) is a commonly used IDL for describing a web service using SOAP. This protocol was very popular in its conception but is nowadays becoming replaced by other solutions such as REST.
• Representational State Transfer (REST) is an architectural style that defines an interface as a means of accessing and manipulating well-identified resources, using HTTP as the transport protocol and a set of methods for reading and writing resource state. REST is praised for its simplicity, performance, scalability and reliability. In the scope of web applications, client modules for consuming RESTful services can be easily implemented without the need for complex external libraries.

Defining an API is very important for software reusability, to ensure that developers allow their services to be integrated in third-party applications. In the biomedical domain, besides the existence of REST web services, use of well-defined standards and vocabulary is also crucial.

**Examples**:

Web service facilities are generally included in software development toolkits and for several programming languages.

### Test-Driven Development (TDD) and Continuous Integration (CI)

**Summary**:

The Test-Driven Development methodology is a software development technique based on short cycles. The basic idea is that the developer creates a set of test cases and writes those test cases to ensure a specific use case. A set of assertions should be established in each test, helping developers to better identify the requirements for each component of the software. As a complement to TDD, Continuous Integration (CI) is a development practice that automates the build, allowing teams to detect early problems.

**Process description**:

In a software development journey, there are often several strategies to bug fixing, and changing the behaviour of modules may introduce problems in other parts of the software. There are three strategies that could be used to tackle the issue:

• Unit and integration tests - Tests written by the programmer to verify if that particular part of the code respects the contract, i.e. what the input and the output is. Integration tests are often built to verify if the different pieces of system work together.
• Continuous integration – A practice that incorporates automatic builds, and allows the teams to detect problems earlier.
• TDD - The practice of writing the tests before writing the code.

TDD can be applied not only with unit tests but also with interfaces. To develop unit tests for the core of the application, it depends on the programming language. The methodology is simple, but application might be more complex. There is always a trade-off between the overhead it introduces and its benefit, so it can be adapted according to specific needs, e.g. validation of critical processes, as is common in the biomedical domain. TDD allows writing of code that automatically verifies if the produced output of an algorithm is as expected ^24^. These tests can be used at any time, allowing to better deal with future changes in code, and saving time in future updates.

TDD and CI make the development process smoother, more predictable and less risky, even in advanced stages of the software lifecycle. Additionally, bugs can be traced and solved sooner, as they are continuously introduced into the project code. CI proposes the following set of development guidelines:

• Do not check in on a broken build;
• Always run all commit tests locally before committing;
• Commit your changes frequently (at least once a day);
• Never go home with changes to commit;
• Never go home on a broken build;
• Always be prepared to revert to the previous revision;
• Take responsibility for all breakages that result from your changes;
• Fix broken builds immediately.

**Examples**:

An example of a tools that can be used for TDD is JUnit for java. To test web interfaces, there is the nightwatch.js tool, amongst others. For CI there are tools such as Jenkins, Travis-CI or TeamCity.

### Documentation

**Summary**:

Documentation is one of the most important aspects of long-term software development. Building comprehensive documentation is very important for software reusability and maintenance, helping to mitigate the arrival/departure of team members Nevertheless, biomedical research software is often born based on experiments and scripts, and researchers are often not willing to document all processes and source code.

**Process description**:

High-level requirements intend to depict what the system “will be”, rather than what it “will do”. The emphasis is therefore on non-functional or business requirements. As the project evolves, these requirements will be progressively more detailed, and eventually converge with low-level requirements. Use case analysis is important for any development project, and it is a task usually shared with end-users. It is important to choose a simple and comprehensive use case template, and sometimes a first iteration with a key user can help refine it before distributing the template among all users.

Other technical documentation needs are mostly related to the project set-up, where a wiki system can be used for storing dispersed information in a controlled environment where everyone is able to edit/comment. This repository can include use cases, architecture/database diagrams, user interface mock-ups, and any project-related documents.

Last but not least, inline source code documentation is very important to define and explain the different parts of the source code, making it easier for the programmers when they need to add extra features or fix bugs. The code must be self-explanatory using an adequate name convention. The inline source code documentation must describe what the code does, how it works, and, when applicable, how it can be integrated with other pieces of code. Nowadays specific and automatic API generation documentation tools allow creation of easy to read documentation based on inline source code documentation.

**Examples**:

For general documentation, Markdown or Sphinx ^3^ (also used for Python) can be used. For Java language, there is Javadoc, while other languages have their own documentation strategy that can be followed. For software specification and requirement analysis there are several templates in OpenUP ^4^ (Open Unified Process) from Eclipse Foundation.

### Software distribution

**Summary**:

Web-based solutions can be deployed in web servers, which makes life a lot easier for the application's end-users who do not need to deal with local installation. It is essential to handle updates smoothly without disrupting the quality of service provided.

**Figure 3.**
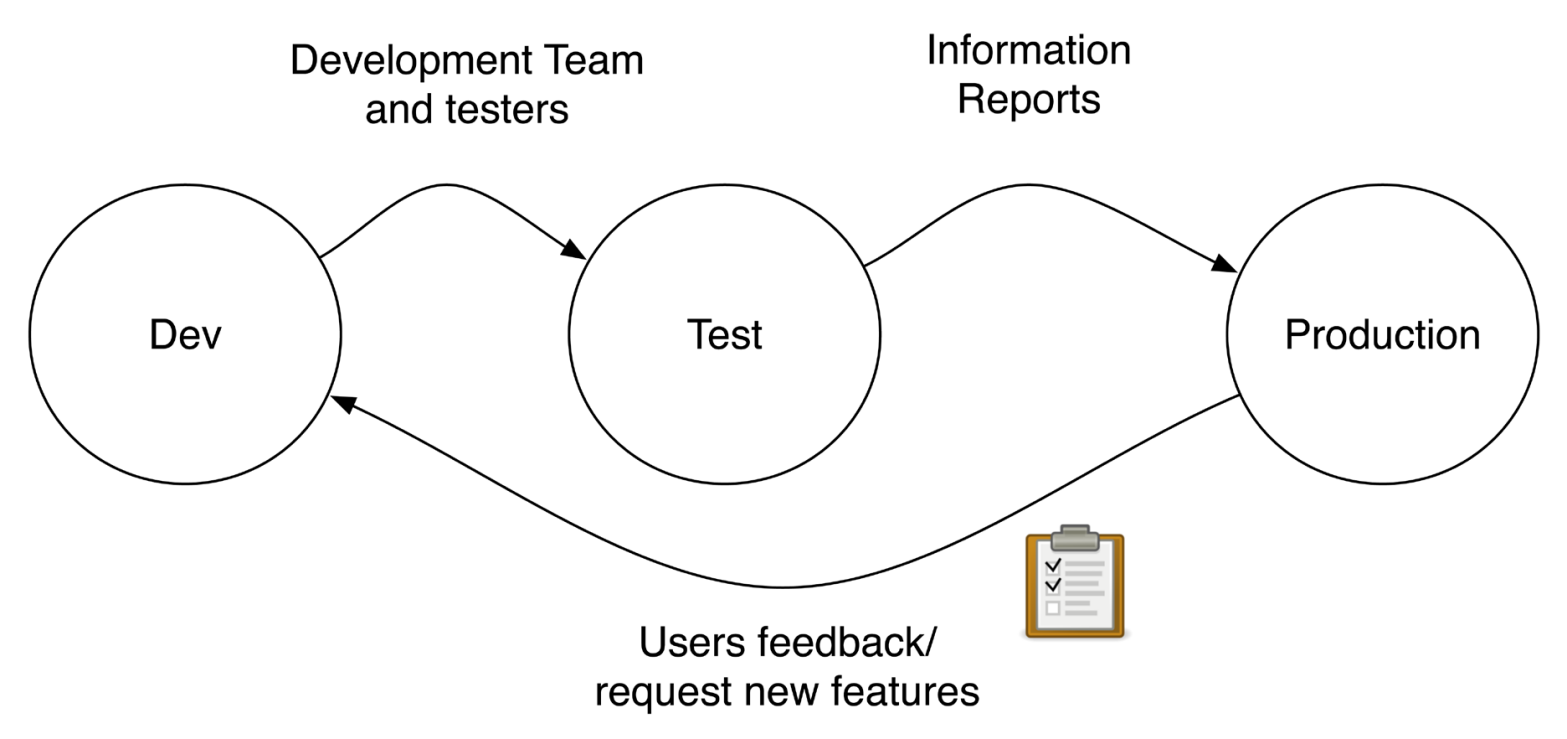
The deployment of each new release should follow three release management steps: development, testing and production.

**Process description**:

The deployment stage of each new release must not be performed in the production environment. It should follow three release management steps: development, testing and production ( Figure 3). These distinct stages have similar conditions and they are deployed over different servers. Also, the production data is replicated in these environments to guarantee that the deployment will be safely performed. Software engineers will often perform the development deployment and test the new features in this environment. When this milestone is reached, the release is performed and updated in the test stage. This version will be passed to a group responsible for testing, gathering feedback and feature enhancement. Once it has passed this stage, the final release will go into production to be used by the end-users.

**Examples:**

This is an organizational guideline, so no special tools are needed. Nevertheless, there are auxiliary tools that help the deployment and distribution process, mainly when the applications require complex setup tools. For example, it is possible to use software containers like Docker ^5^ to distribute complex software and help deploy it, ensuring the whole community can run the software ^25– 27^.

### Licensing

**Summary:**

Licensing and copyright attribution is a subject that should be addressed from the very beginning of the project. The goal is to clarify the terms that will regulate future use of the software – e.g. commercial, free use, open source. Open source software is currently a trend, even in bigger companies, as a way to credit the authors and promote work dissemination and collaborative development. Several kinds of licenses are available to regulate these relationships, although an individual disclaimer can be written. A commonly used license is the Free and Open Source Software (FOSS) license, which allows the product to be modified and redistributed without having to pay the original author.

**Process description:**

The license should be stated clearly on the project’s front page and in the root of the source code. The full license text can be included here in a file called *COPYING* or *LICENSE*, following the standard format.

The copyrights should be assigned together with the license. The common nomenclature adds the year and the organization owning the copyright: *Copyright (C) <year><name of organization>.* The year specification may be a range, such as 2014–2016, to restrict the copyright to a period of time ^28^. This line should be included in the headers of all source code files, together with a short license.

**Examples:**

There are different types of open source licenses, that come with different conditions and restrictions. We will list the most commonly used open source licenses:

• **BSD License –** It is the most permissive FOSS license. Users that re-use the code can do whatever they want, except in the case of redistributing source or binary, where they must always retain the copyright notice.
• **Apache Public License 2.0 –** This license is very permissive. It allows the licensed source code to be used in open-source and also in closed-source software.
• **GNU GPL –** This license is restrictive. The users of the licensed system are free to use the licensed system without any usage restrictions; analyze it and use the results of the analysis (the source code must be provided and cannot be hidden); redistribute unchanged copies of the licensed system, and also modify and redistribute modified copies of the licensed system
• **GNU LGPL –** It is trade-off between the restricted GNU GPL and the permissive BSD. LGPL assumes that a library licensed under LGPL can be used in a non-GPL application. All the changes applied to the LGPL library must remain under LGPL. It assumes that all copyrights reversed on source code files, and not on the whole program.

## Conclusion and future directions

In the biomedical domain, many new code scripts, algorithms, tools and services are currently being developed on a worldwide scale. However, the reuse of some of these software solutions outside the research lab is being hindered by them not following consolidated software developing methodologies. Early adoption of these methodologies is important in the development of biomedical tools so that they can reach a greater number of users; not only researchers but also healthcare professionals. During the development and distribution processes it is very important to involve end-users, to collect as much feedback as possible and create effective solutions during the development process.

We described a set of recommendations targeted at biomedical software developers aimed at achieving a good balance between fast prototyping, and robustness and long term maintenance. It is important to keep in mind that these recommendations are quite general and may not fit all cases, so adaptations may be required. We hope they can help biomedical researchers to reorganize their workflow, make their tools more visible, allow reproducibility of their research, and most importantly, that the outcome of that research can be more easily translated into daily clinical practice.

## Notes

^1^ http://nvie.com/posts/a-successful-git-branching-model/

^2^ https://www.w3.org/TR/soap

^3^ http://www.sphinx-doc.org/

^4^ http://epf.eclipse.org/wikis/openup/

^5^ https://www.docker.com/
