# Supplementary material for: General guidelines for biomedical software development
Source: F1000Res. 2017 Jul 12;6:273. Originally published 2017 Mar 15. [Version 2] doi: 10.12688/f1000research.10750.2 (PMC5383938; doi:10.12688/f1000research.10750.2)
Supplement: Peer review history

# Peer review: Referee response for version 1

**DOI:** 10.5256/f1000research.11591.r21000

**Published:** 2017-04-05

**Authors:** Victor Maojo

**Affiliations:** 1 Biomedical Informatics Group, Artificial Intelligence Department, Universidad Politecnica de Madrid, Madrid, Spain

This is a timely report, given the proliferation of all types of biomedical informatics applications (from medical apps to laboratory or even complex clinical ones) delivered by software developers that do not follow even simple criteria of solid software engineering. In fact, many of these applications are built to carry out quite simple computational tasks (even, many times, quite successfully) but without a sound rigorous computing basis, and then are prone to multiple subtle errors or they lack standardized approaches and interoperability capacities. Besides the interest of the topic, the paper is well written, with a solid analysis of the topic, useful recommendations and a selected reference section, which can be very helpful to a broad range of readers, from public health informaticians to bioinformaticians. Below are some comments.

  

1. Although the authors are usually careful with this issue, readers outside the field may have some problems to understand the differences between medical informatics, bioinformatics, computational biologists and biomedical informatics. Sometimes the words are used in an interchangeable way in the paper, but this may lead to confusion. Some explanation might be necessary.
2. When the authors refer to “focus on decision support, NLP, information retrieval and EHRs” they mix techniques and a concrete system (EHR). They should explicit what technique they refer for EHRs.
3. The authors begin to address, apparently, biomedical informatics (thus, including public health and clinical topics) but they focus later in bioinformatics, which I believe it is the best target for the paper. Differences are usually significant between clinical (for instance, EHRs, with many big software companies dedicated to this field). Some example may be useful.
4. Besides the provided hyperlink, some reference should be added for the MedBioinformatics project and a brief description.
5. The software engineering guidelines suggested are of interest, but some additional brief comparison and similarities/differences with established methodologies (besides what is presented for Test-driven) may provide additional insight.
6. As mentioned above, some brief, real example carried out by the authors may add some information of interest, pointing out actual problems and possible approaches. In fact, the paper is quite generic, but some specific case/application in the biomedical domain can be of interest.
7. Some differences may be pointed out when software developers work for an academic thesis or project, compared to a software company? Some comment may be of interest, too. In fact, many tools are quite simple, for a single task, and not intended for broader scenarios, where interoperability is necessary. Some recommendations could differentiate both cases.
8. For this reviewer, the paper may require some more explanation about design and prerequisites aspects, which are quite important, and some concrete example, but this is quite personal and the decision should be up to the authors.

I have read this submission. I believe that I have an appropriate level of expertise to confirm that it is of an acceptable scientific standard.

# Response

**Published:** 2017-07-07

**Authors:** Luis Bastiao Silva

Thank you for the positive assessment and helpful recommendations. We will answer point by point for your comments: 

1) We agree, this discussion is important. We have included in this revision two new references where the explanation of the different fields is well addressed. 

2) Done

3) Indeed, this is true and we tried to make it more clear along the article. Moreover, we included new references that discuss this issue in detail.

4)  Thank you for highlighting this. A brief introduction to the project is now provided.

5/6) Indeed, we agree with this remark. However, since these recommendations result from the experience of several software projects, where many concrete use cases were explored, we also feel that detailing those could be out of scope of the article. 

7) Thank you for raising this, which is indeed a very important remark. We have now discussed this in more detail in the introduction section.

8) We agree with your remark. The design and prerequisites aspects are briefly addressed in the documentation process. We changed this section to highly better these issues.

# Peer review: Referee response for version 1

**DOI:** 10.5256/f1000research.11591.r21001

**Published:** 2017-04-03

**Authors:** João P. G. L. M. Rodrigues

**Affiliations:** 1 Department of Structural Biology, School of Medicine, Stanford University, Stanford, CA, USA

This review focuses on an important topic in modern bioinformatics: good practices for software development. As the authors note, there is a growing body of data derived from experimental studies that requires automated analysis. The analysis is often carried out using custom software, written by first-time or inexperienced programmers, and results in unsupported, sub-optimal, or duplicated code. As the authors also mention, several groups have tried to put forward a collection of tips and guidelines to help researchers in developing ‘proper’ software. This review offers a similar set of guidelines, targeted specifically at the field of biomedical informatics, and draws on the experience of the authors on building their own tools.

 The suggestions cover seven topics, from management to in-depth software development tips, and do a very good job at explaining their importance and their take on what constitutes a good approach. The authors also give very good examples of software tools to help readers setup a development environment. These range from the usual ‘use GitHub’ to TravisCI, Sphinx, and Docker. One suggestion would be to integrate some of these tools in Figure 1, to give readers a visual cue where these tools fit in each topic/step. The authors also provide a very nice summarized view of the release process, namely licensing and distribution (e.g. using Docker), and the follow-up maintainance.

 There is one less positive aspect of this review, which is anyway transversal to most such attempts at ‘guidelines’ for bioinformatics software development. As the authors note, most of these tools are created to solve one very specific problem, or process a very specific dataset. These are not amenable to test-driven development, or to continuous integration. More importantly, most of the authors of these tools/scripts are biologists, not programmers, which usually translates to a lack of interest in proper programming etiquette. Thus, I believe that it is important to show and teach such users very very simple programming rules, namely about how to make their code readable for others. For example, in the Python world, a simple recommendation to use ‘flake8’ to check for PEP8 coding standards and an editor (e.g. Atom, Sublime) that can do real-time code checks (typos, unused variables, indentation issues, etc). There is no need to suggest quasi-professional IDEs, as these will likely scare users away!

 All in all, as a biologist doing bioinformatics and doing his best to follow proper software guidelines, I find reviews like this one very important to the field. They should probably feature in a ‘starting package’ to new PhD students in many labs. As an added suggestion, the authors could think of putting these guidelines in practice and follow up with a simple workshop/tutorial series, a la software carpentry, even if in webinar format.

I have read this submission. I believe that I have an appropriate level of expertise to confirm that it is of an acceptable scientific standard.

# Response

**Published:** 2017-07-07

**Authors:** Luis Bastiao Silva

Much obliged for your assessment and recommendations. We have redrawn Figure 1 following your suggestion. Regarding the second point, we recognised the importance of the subject and how recommendations vary according each developer/research profile and even programming language. For beginners or sporadic developers, most of the recommendation may not apply. However, this type of review creates the awareness of developing for the community, not just for ourselves. Finally, regarding last comment, indeed, guidelines for a new comers is good idea. We think this can be done at the institution level, since different methodologies may be used locally.
